# A Relação entre Regurgitação Mitral e Implante Transcateter de Válvula Aórtica: um Estudo de Acompanhamento Multi-Institucional

**DOI:** 10.36660/abc.20190772

**Published:** 2021-06-08

**Authors:** Luciana de Cerjat Bernardes P. da Cunha, Enio Eduardo Guerios, Claudio Leinig Pereira da Cunha, Luiz A. Carvalho, Pedro Lemos, Rogério Sarmento-Leite, Alexandre A. Abizaid, José Antonio Mangione, Adriano Dourado Oliveira, Alexandre Siciliano, Vinicius Esteves, Fábio Sândoli de Brito

**Affiliations:** 1 Universidade Federal do Paraná Hospital de Clínicas CuritibaPR Brasil Universidade Federal do Paraná - Hospital de Clínicas - UFPR, Curitiba , PR - Brasil; 2 Hospital Pró-Cardíaco Rio de JaneiroRJ Brasil Hospital Pró-Cardíaco , Rio de Janeiro , RJ - Brasil; 3 Hospital Israelita Albert Einstein São PauloSP Brasil Hospital Israelita Albert Einstein , São Paulo , SP - Brasil; 4 Instituto de Cardiologia Porto AlegreRS Brasil Instituto de Cardiologia , Porto Alegre , RS - Brasil; 5 Hospital Beneficência Portuguesa de São Paulo São PauloSP Brasil Hospital Beneficência Portuguesa de São Paulo , São Paulo , SP - Brasil; 6 Hospital Santa Izabel SalvadorBA Brasil Hospital Santa Izabel , Salvador , BA - Brasil; 7 Rede D’Or São Luiz São PauloSP Brasil Rede D’Or São Luiz , São Paulo , SP - Brasil; 8 Universidade de São Paulo Instituto do Coração São PauloSP Brasil Universidade de São Paulo Instituto do Coração , São Paulo , SP - Brasil; 9 Hospital Sírio-Libanês São PauloSP Brasil Hospital Sírio-Libanês , São Paulo , SP - Brasil

**Keywords:** Insuficiência da Valva Aórtica, Insuficiência da Valva Mitral, Implante Transcateter da Válvula Aórtica, Epidemiologia, Análise de Sobrevida, Ecocardiografia/métodos

## Abstract

**Fundamento:**

A regurgitação mitral (RM) é prevalente em pacientes submetidos a implante transcateter de válvula aórtica (TAVI). Há algumas controvérsias sobre o impacto prognóstico da RM na sobrevida de pacientes submetidos a TAVI.

**Objetivo:**

Examinar a relação entre TAVI e RM em uma população de pacientes do Registro Brasileiro de TAVI.

**Métodos:**

Setecentos e noventa e cinco pacientes do Registro Brasileiro de TAVI foram divididos na linha de base, alta e acompanhamento de acordo com o grau da RM da maneira seguinte: ausente/leve (RMAL) ou moderado/grave (RMMG). Eles foram subsequentemente reagrupados de acordo com as mudanças imediatas e tardias na gravidade da RM após TAVI da maneira seguinte: RM sem mudança, melhora ou piora. Foram analisados os preditores e o impacto prognóstico na linha de base, bem como as mudanças na gravidade da RM. A significância estatística foi estabelecida em p < 0,05.

**Resultados:**

RMMG basal estava presente em 19,3% dos pacientes e foi um preditor de aumento da mortalidade tardia. Imediatamente após o TAVI, 47,4% dos casos melhoraram para RMAL, previsto por uma pontuação mais alta da *Society of Thoracic Surgeons* e um grau mais alto de regurgitação aórtica basal. No acompanhamento, 9,2% dos casos de RMAL pioraram para RMMG, enquanto 36,8% dos casos de RMMG melhoraram para RMAL. Fração de ejeção do ventrículo esquerdo (FEVE) mais baixa na linha de base e melhora na FEVE durante o acompanhamento foram preditores de melhora da RM. Piora progressiva da RM no acompanhamento foi um preditor independente de maior mortalidade tardia após TAVI (p = 0,005).

**Conclusões:**

A RMMG na linha de base é um preditor de mortalidade tardia após TAVI. FEVE mais baixa e melhora na FEVE durante o acompanhamento são preditores de melhora da RM após TAVI. A pior progressiva da gravidade da RM durante o acompanhamento é um preditor independente de mortalidade tardia; isto é um achado raro na literatura.

## Introdução

Aproximadamente dois terços dos pacientes com estenose aórtica (EAo) sintomática grave e indicação para cirurgia de troca valvar apresentam algum grau de regurgitação mitral (RM) ^1^ e, em alguns casos, indicação de cirurgia de dupla troca valvar. ^2^ Para pacientes submetidos à troca isolada da válvula aórtica, RM moderada ou grave pode estar associada a maiores taxas de mortalidade, insuficiência cardíaca congestiva e subsequente cirurgia da válvula mitral. ^3^

Para pacientes com EAo e RM graves para os quais a cirurgia não é a escolha terapêutica ideal, implante transcateter de válvula aórtica (TAVI) pode ser uma opção apropriada. ^1 , 2^ Visto que, em alguns pacientes, uma redução de grau pode ser esperada ou pode ser indicada subsequente intervenção transcateter da válvula mitral, a RM geralmente não é tratada neste cenário. ^1 , 4^ Porém, no caso de cirurgia aórtica isolada, a gravidade da RM pode diminuir, permanecer inalterada ou mesmo aumentar após TAVI. ^1 , 5^ Embora muitos estudos demonstrem consistentemente que a RM importante na linha de base está associada a resultados piores, ^4 , 6^ informações sobre as implicações prognósticas de mudanças na gravidade da RM após TAVI são escassas. ^7^

O objetivo do presente estudo foi o de examinar a relação entre TAVI e RM em uma população de pacientes do Registro Brasileiro de TAVI. ^8^ A nossa hipótese foi que RM moderada/grave (RMMG) na linha de base e deterioração progressiva da RM influenciam o prognóstico de TAVI.

## Métodos

### Pacientes

O Registro Brasileiro de TAVI multicêntrico é um registro de participação voluntária, realizado desde 2008 pela Sociedade Brasileira de Cardiologia Intervencionista, que agrega os resultados de TAVI realizados em 22 centros de todo o Brasil. Os pacientes foram retrospectiva e prospectivamente incluídos no registro desde o primeiro TAVI realizado no Brasil. O registro foi aprovado pelo Comitê de Ética do Hospital Albert Einstein de São Paulo, em 10 de novembro de 2010, e inserido na Plataforma Brasil (uma base de dados nacional e unificada de registros de pesquisas envolvendo seres humanos). Todos os pacientes prospectivamente incluídos forneceram consentimento esclarecido por escrito.

A indicação de TAVI foi limitada a grupos de pacientes inoperáveis ou de alto risco cirúrgico com EAo sintomática grave ou bioprótese cirúrgica degenerada. O risco de mortalidade cirúrgica foi estimado usando o EuroScore ^9^ e o escore de risco da Society of Thoracic Surgeons (STS). ^10^ Os detalhes, as definições e os resultados de registros parciais foram publicados anteriormente. ^8^

O presente estudo incluiu pacientes tratados entre janeiro de 2008 e janeiro de 2015. Foram excluídos da análise os pacientes previamente submetidos a cirurgia da válvula mitral e os pacientes que não apresentavam registros ecocardiográficos pré- e pós-intervenção adequados. O acompanhamento foi realizado nas consultas médicas com exames ecocardiográficos; o último ecocardiograma de acompanhamento foi usado para comparar com os exames de linha de base e de alta.

### Procedimento de TAVI

Foi realizado o TAVI usando próteses CoreValve (Medtronic, Minneapolis, MN, EUA) por acesso transfemoral e transubclávia, Sapien XT (Edwards Lifesciences, Irvine, CA, EUA) por acesso transfemoral e transapical, e Inovare (Braile Biomédica, São José do Rio Preto, SP, Brasil) implantado apenas pelo acesso transapical. O procedimento foi realizado de acordo com técnicas de padrão, previamente detalhadas. ^11 - 13^ A escolha do acesso, o tipo de anestesia (geral ou sedação) e o uso da ecocardiografia transesofágica intra-operatória foram deixados a critério do operador. Após a intervenção, foram administrados aspirina (100 mg uma vez ao dia) e clopidogrel (300 mg como dose de ataque e 75 mg uma vez ao dia posteriormente) aos pacientes durante um período mínimo de 30 dias. Um ecocardiograma transtorácico completo dos pacientes foi realizado nos períodos pré-, peri- e pós-intervenção (quando havia vários ecocardiogramas, foi incluído o último). A gravidade da RM foi definida como ausente, leve, moderada ou grave de acordo com as recomendações da American Society of Echocardiography, integrando parâmetros estruturais, Doppler e quantitativos. ^14^

Os dados clínicos e os ecocardiogramas dos pacientes foram analisados na linha de base, na alta hospitalar e no acompanhamento tardio (tempo médio de acompanhamento de 16,6 meses). Em cada um desses períodos, os pacientes foram separados em dois grupos, de acordo com o grau de RM. Um grupo incluiu pacientes com RM ausente ou leve (RMAL) e o outro incluiu aqueles com RMMG, conforme descrito em estudos anteriores. ^2 , 15^ Subsequentemente, os pacientes foram reagrupados de acordo com a mudança na gravidade da RM após TAVI, ao comparar os períodos da linha de base, da alta e do acompanhamento, da maneira seguinte: pacientes que não apresentaram alteração no grau de RM, aqueles com piora da RM (de RMAL para RMMG) e aqueles com melhora da gravidade de RM (de RMMG para RMAL). Foram identificados os preditores clínicos e ecocardiográficos de melhora/piora da RM e foi analisada a relação entre as mudanças no grau de RM e as taxas de mortalidade.

### Análise estatística

Foram realizadas as análises estatístcas com IBM SPSS Statistics para Windows, Versão 20.0 (IBM Corp, Armonk, NY, EUA). As variáveis contínuas foram expressas como média e desvio padrão ou mediana e intervalo, enquanto as variáveis categóricas foram expressas como frequências e porcentagens. O teste de Kolmogorov-Smirnov foi usado para verificar a normalidade dos dados; a normalidade da distribuição dos dados foi aceita para a maioria das variáveis, sem comprometer as demais análises. As associações de variáveis categóricas entre os grupos foram avaliadas por meio do teste qui-quadrado de Pearson. As variáveis contínuas foram analisadas usando o teste *t* de Student para amostras independentes ou o teste não paramétrico de Mann-Whitney para comparar grupos definidos pelo grau de RM basal (RMAL ou RMMG). A análise de variância (ANOVA) unilateral ou o teste não paramétrico de Kruskal-Wallis foi usado para comparar os grupos definidos pelas mudanças de RM (sem mudança, piora ou melhora). A probabilidade de sobrevida foi estimada por curvas de Kaplan-Meier. Para analisar o efeito das mudanças de RM no tempo de sobrevida, modelos de regressão de risco proporcional de Cox não ajustados e ajustados foram adaptados, incluindo covariáveis com p < 0,05 nos modelos não ajustados. Os modelos finais foram avaliados pelo método da razão de verossimilhança stepwise e backward, considerando-se o valor de p < 0,05 para os critérios de inclusão e exclusão. A razão de risco (HR, da sigla em inglês para *hazard ratio* ) e os intervalos de confiança (IC) de 95% foram apresentados para os modelos finais. A significância estatística foi estabelecida em p < 0,05.

## Resultados

### Características de linha de base dos pacientes

**Figura 1:**
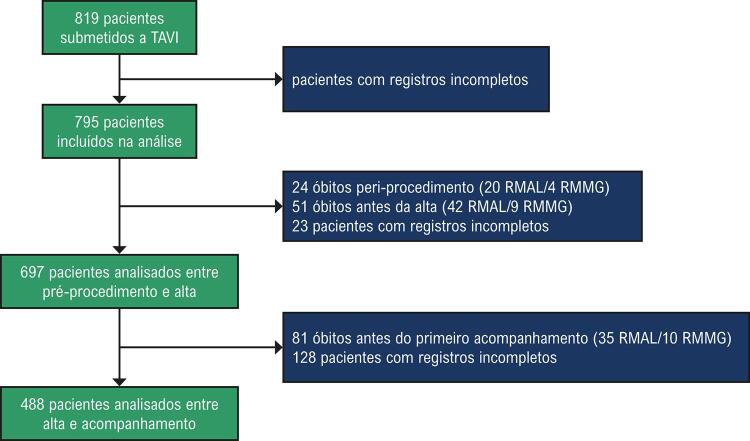
– Diagrama de fluxo dos pacientes. Este fluxograma especifica a taxa de mortalidade dos grupos RMAL e RMMG. É possível observar que a taxa de mortalidade antes da alta inclui a mortalidade peri-procedimento. Os “registros incompletos” estão relacionados à ausência de ecocardiogramas adequados para análise. RMAL: regurgitação mitral ausente/leve; RMMG: regurgitação mitral moderada/grave.

Dos 819 pacientes incluídos no Registro Brasileiro de TAVI, 795 pacientes foram incluídos nesta análise. Um diagrama de fluxo dos pacientes é apresentado na Figura 1 e a Tabela 1 detalha as características clínicas de linha de base dos pacientes de acordo com o seu grau de RM de linha de base. Antes do procedimento, a RM era ausente/leve em 642 pacientes (80,7%) e moderada/grave em 153 pacientes (19,3%). Os pacientes com RMMG eram mais velhos e apresentavam mais comorbidades (insuficiência renal, níveis mais baixos de hemoglobina, hipertensão pulmonar, fibrilação atrial, implante de marca-passo anterior, graus mais avançados de insuficiência cardíaca), escores de risco cirúrgico mais elevados, frações de ejeção mais baixas, diâmetros diastólicos de VE maiores, mais regurgitação aórtica grave, áreas valvares aórticas menores e gradientes aórticos mais baixos.

**Tabela 1:** Características de linha de base dos pacientes e comparação de grupos definidos por disfunção de RM na linha de base (n = 795)

Característica	População inteira (n = 795)	De acordo com disfunção RM na linha de base		valor p*

		Ausente/leve (n = 642)	Moderada/grave (n = 153)	
Idade (anos)	81,5 ± 7,3	81,2 ± 7,5	83,1 ± 6,6	0,002
Sexo masculino	389 (48,9)	313 (48,8)	76 (49,7)	0,838
Doença arterial coronariana	465 (58,4)	375 (58,4)	90 (58,8)	0,926
Infarto do miocárdio prévio	117 (14,7)	99 (15,4)	18 (11,8)	0,251
Doença vascular periférica	136 (17,1)	118 (18,4)	18 (11,8)	0,051
Acidente vascular cerebral/AIT	63 (7,9)	50 (7,8)	13 (8,5)	0,771
Diabetes	253 (31,8)	206 (32,1)	47 (30,7)	0,744
Hipertensão arterial sistêmica	601 (75,5)	484 (75,4)	117 (76,5)	0,780
Insuficiência renal	615 (77,3)	485 (75,5)	130 (85,0)	0,012
Marca-passo pré-procedimento	81 (10,2)	57 (8,8)	24 (15,6)	0,012
Hemoglobina (mg/dl)	11,8 ± 1,7	11,8 ± 1,8	11,5 ± 1,6	0,045
Hipertensão pulmonar	176 (22,1)	133 (20,7)	43 (28,1)	0,048
Classe funcional NYHA III ou IV	648 (81,5)	511 (79,6)	137 (89,5)	0,004
Fibrilação atrial	106 (13,3)	78 (12,3)	28 (18,5)	0,044
Mortalidade EuroScore	16 ( 17,6)	15,2 (16,6)	21,1 (17,5)	0,001
Mortalidade STS	7,2 (10,5)	6,6 (9,9)	10,9 (12)	<0,001
Regurgitação aórtica basal moderada/grave	95 (11,9)	60(10,9)	35(23,0)	<0,001
Valvoplastia aórtica por balão prévia	50 (6,2)	36 (5,6)	14 (9,2)	0,105
FE basal (%)	58,7 ± 14,9	60,1 ± 14,4	53,2 ± 16,0	<0,001
Diâmetro diastólico basal do VE (mm)	50,8 ± 9,4	50,2 ± 8,8	53,4 ± 10,3	0,001
Área basal da válvula aórtica Área basal da válvula aórtica (cm ^2^ )	0,66 ± 0,19	0,67 ± 0,19	0,63 ± 0,19	0,016
Gradiente aórtico médio basal (mmHg)	49,3 ± 16,0	50,1 ± 15,7	46,3 ± 16,5	0,010
Gradiente aórtico máximo basal (mmHg)	81,0 ± 24,8	82,3 ± 24,6	76,0 ± 25,0	0,005

Resultados descritos por frequência (porcentagem), média ± desvio padrão ou mediana (intervalo interquartil). *Teste t de Student para amostras independentes, teste não paramétrico de Mann-Whitney (variáveis quantitativas) ou teste do qui-quadrado (variáveis categóricas), p < 0,05. AIT: ataque isquêmico transitório, FE: fração de ejeção, NYHA: New York Heart Association, RM: regurgitação mitral, STS: Society of Thoracic Surgeons, VE: ventrículo esquerdo.

Foram implantadas as próteses CoreValve em 597 pacientes (73%) por acessos transarteriais, Sapien XT em 200 pacientes (24%) (3 por via transapical e 197 por via transarterial) e Inovare em 22 pacientes (3%) por acessos transapicais. No total, 770 pacientes receberam as próteses por via transarterial, enquanto 25 foram por via transapical. Setecentos e setenta e nove pacientes (98%) receberam próteses para EAo grave nativa e 16 (2%) receberam próteses válvula-em-válvula para biopróteses cirúrgicas degeneradas.

### Preditores de mortalidade tardia

De acordo com o modelo de regressão de Cox ajustado, a doença vascular periférica (HR 1,6; IC 95%, 1,11-2,32; p = 0,012), valvoplastia aórtica por balão anterior (HR 1,97; IC 95%, 1,25-3,11; p = 0,004), e RMMG na linha de base (HR 1,50; IC 95%, 1,05-2,14; p = 0,027) foram preditores independentes basais de mortalidade tardia, com tempo médio de acompanhamento de 16,6 meses e acompanhamento médio de 12,4 meses (primeiro quartil: 2,6 meses e terceiro quartil: 24,7 meses) nesta população.

### Mudanças na gravidade da RM: pré-intervenção versus alta

**Figura 2:**
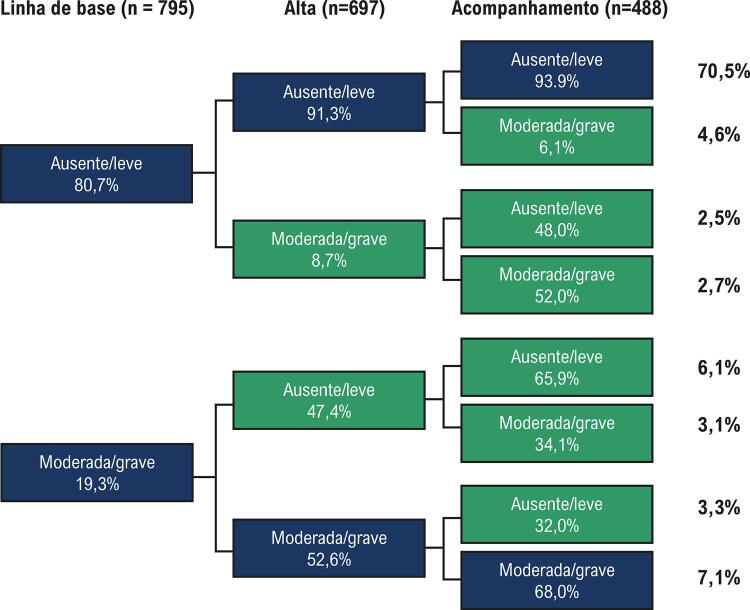
– Mudanças na gravidade da regurgitação mitral (RM): linha de base, alta e períodos de acompanhamento. Isso inclui pacientes com dados ecocardiográficos completos para todos os três períodos. Linha de base: n = 795; alta: n = 697; acompanhamento: n = 488. **Variação do grau de RM ao comparar a linha de base com o último acompanhamento para toda a população, excluindo óbitos e registros incompletos.

Após a intervenção, o grau de RM foi comparado entre a linha de base e a alta em um total de 697 pacientes. O TAVI não alterou o grau de RM em comparação com a linha de base em 83,8% (n = 584) dos pacientes. A gravidade da RM piorou após TAVI em 8,7% (n = 49) dos pacientes com RMAL na linha de base, mas melhorou em 47,8% (n = 64) daqueles com RMMG na linha de base ( Figura 2 ).

Houve maior prevalência de insuficiência renal em pacientes cujo grau de RM piorou após TAVI (p = 0,022). Na análise univariada, escore STS mais alto (p = 0,013) e regurgitação aórtica basal mais grave (p = 0,010) foram preditores de melhora na gravidade da RM. Outros dados ecocardiográficos basais, bem como alterações de parâmetros, como a fração de ejeção do ventrículo esquerdo (FEVE) e o gradiente aórtico entre a linha de base e a alta, não foram associados a melhora ou piora da gravidade da RM após TAVI ( Tabela 2 ).

**Tabela 2:** – Comparação de grupos definidos por mudanças na gravidade da RM: linha de base versus alta após TAVI (n = 697)

Característica	Mudanças na gravidade da RM na linha de base versus alta			valor p*

	Sem mudança n = 584	Piora n = 49	Melhora n = 64	
Idade (anos)	81,3 ± 7,5	82,4 ± 5,5	81,9 ± 6,7	0,559
Sexo masculino	294 (50,3)	21 (42,9)	31 (48,4)	0,590
Doença arterial coronariana	332 (56,8)	35 (71,4)	38 (59,4)	0,136
Infarto do miocárdio prévio	90 (15,4)	7 (14,3)	7 (10,9)	0,629
Doença vascular periférica	106 (18,2)	10 (20,4)	6 (9,4)	0,184
Acidente vascular cerebral/AIT	49 (8,4)	4 (8,2)	5 (7,8)	0,986
Diabetes	187 (32,0)	13 (26,5)	20 (31,2)	0,728
Hipertensão arterial sistêmica	429 (73,5)	40 (81,6)	51 (79,7)	0,279
Insuficiência renal	444 (76,0)	44 (89,8)	55 (85,9)	0,022
Hemoglobina (mg/dl)	11,8 ±1,8	12,0±1,7	11,5±1,5	0,374
Hipertensão pulmonar	130 (22,3)	8 (16,3)	17 (26,6)	0,431
Classe funcional NYHA III ou IV	471 (80,7)	39 (79,6)	55 (85,9)	0,570
Fibrilação atrial	73 (12,7)	8 (16,3)	10 (15,6)	0,642
Mortalidade EuroScore	15,6 (17)	17,4 (15,7)	21,1 (17,9)	0,124
Mortalidade STS	6,9 (10,2)	9,5 (14,5)	11,5 (12,1)	0,013
Regurgitação aórtica basal moderada/grave (AR)	69 (11,9)	5 (10,9)	16 (25,4)	0,010
Valvoplastia aórtica por balão prévia	33 (5,7)	3 (6,1)	7 (10,9)	0,309
FE basal (%)	59,2 ± 15,0	55,3 ± 15,5	57,3 ± 14,7	0,160
Diâmetro diastólico basal do VE (mm)	50,8 ± 9,0	51,2 ± 11,0	52,4 ± 10,0	0,430
Área basal da válvula aórtica (cm ^2^ )	0,67 ± 0,19	0,67 ± 0,17	0,63 ± 0,20	0,360
Gradiente aórtico médio basal (mmHg)	49,5 ± 16,0	46,3 ± 12,6	49,5 ± 19,2	0,434
Gradiente aórtico máximo basal (mmHg)	80 (33)	75 (34,5)	78 (37,5)	0,324
FE, diferença entre a linha de base e a alta (%)	1 (10)	1 (16,3)	3 (10)	0,314
Gradiente aórtico médio, diferença entre a linha de base e a alta (mmHg)	-39,6 ± 16,1	-39,7 ± 12,9	-37,5 ± 23,0	0,686
Gradiente aórtico máximo, diferença entre a linha de base e a alta (mmHg)	-63,1 ± 24,9	-60,3 ± 22,3	-56,6 ± 34,2	0,174

Resultados descritos por frequência (porcentagem), média ± desvio padrão ou mediana (intervalo interquartil). *ANOVA unilateral, teste não paramétrico de Kruskal-Wallis (variáveis quantitativas) ou teste do qui-quadrado (variáveis categóricas), p < 0,05. AIT: ataque isquêmico transitório, FE: fração de ejeção, NYHA: New York Heart Association, RM: regurgitação mitral, STS: Society of Thoracic Surgeons, TAVI: implante transcateter de válvula aórtica, VE: ventrículo esquerdo.

### Mudanças na gravidade da RM: alta versus acompanhamento

Após a alta, o acompanhamento clínico e ecocardiográfico foi realizado em 488 pacientes, com tempo médio de acompanhamento de 16,6 ± 14,1 meses (acompanhamento mediano: 12,4 meses, primeiro quartil: 2,6 meses e terceiro quartil: 24,7 meses). Em comparação com a alta, não houve mudanças na gravidade da RM em 86,4% (n = 422) dos pacientes. Apenas 9,2% (n = 38) dos pacientes com RMAL na alta apresentaram graus piores da gravidade da RM, enquanto 36,8% (n = 28) dos pacientes com RMMG na alta apresentaram melhora para RMAL durante o acompanhamento ( Figura 2 ).

A FEVE mais baixa na linha base (p = 0,015) foi um preditor de melhora tardia da gravidade da RM na análise univariada. Além disso, foi observada uma forte tendência de melhora tardia da gravidade da RM em pacientes com melhora da FEVE durante o acompanhamento (p = 0,052, Tabela 3 ). Não foram identificados fatores preditivos de piora tardia da gravidade da RM.

**Tabela 3:** – Comparação de grupos definidos por mudanças na gravidade da RM: alta após TAVI versus períodos de acompanhamento (n = 488)

Característica	Mudanças na gravidade da RM na alta versus acompanhamento (médio = 16,6 meses)			valor p*

	Sem mudança n = 422	Piora n = 38	Melhora n = 28	
Idade (anos)	81,1 ± 7,3	81,7 ± 6,4	83,9 ± 6,6	0,119
Sexo masculino	216 (51,2)	15 (39,5)	13 (46,4)	0,356
Doença arterial coronariana	238 (56,4)	25 (65,8)	19 (67,9)	0,287
Infarto do miocárdio prévio	61 (14,5)	7 (18,4)	6 (21,4)	0,538
Doença vascular periférica	73 (17,3)	6 (15,8)	6 (21,4)	0,830
Acidente vascular cerebral/AIT	27 (6,4)	4 (10,5)	2 (7,1)	0,659
Diabetes	128 (30,3)	13 (34,2)	10 (35,7)	0,755
Hipertensão arterial sistêmica	306 (72,5)	27 (71,1)	21 (75,0)	0,938
Insuficiência renal	323 (76,5)	30 (78,9)	26 (92,9)	0,131
Hemoglobina (mg/dl)	11,8 ± 1,7	11,8 ± 1,7	11,8 ± 2,0	0,968
Hipertensão pulmonar	85 (20,1)	9 (23,7)	11 (39,3)	0,055
Classe funcional NYHA III ou IV	347 (82,2)	28 (73,7)	24 (85,7)	0,365
Fibrilação atrial	50 (12,0)	7 (18,4)	5 (17,9)	0,407
Mortalidade EuroScore	15,2 (15,8)	19,8 (20)	18,4 (21,2)	0,077
Mortalidade STS	7 (10,7)	10,9 (13,2)	10,6 (8,2)	0,254
Regurgitação aórtica basal moderada/grave	54 (13,1)	6 (16,2)	3 (11,1)	0,825
Valvoplastia aórtica por balão prévia	28 (6,6)	3 (7,9)	1 (3,6)	0,744
FE basal (%)	58,6 ± 15,3	59,0 ± 14,5	49,8 ± 16,5	0,015
Diâmetro diastólico basal do VE (mm)	50,6 ± 8,0	51,4 ± 9,0	51,8 ± 8,0	0,569
Área basal da válvula aórtica (cm ^2^ )	0,66 ± 0,19	0,70 ± 0,14	0,62 ± 0,23	0,317
Gradiente aórtico médio basal (mmHg)	50,5 ± 16,3	46,0 ± 14,4	45,7 ± 14,4	0,104
FE na alta (%)	60,4 ± 13,4	61,4 ± 12,7	55,3 ± 15,3	0,117
Diâmetro diastólico do VE na alta (mm)	50,4 ± 9,0	51,8 ± 9,0	51,6 ± 8,0	0,642
Gradiente aórtico médio na alta (mmHg)	10,2 ± 6,1	9,2 ± 7,9	7,6 ± 3,7	0,131
Gradiente aórtico máximo na alta (mmHg)	18 (11)	15,5 (12,5)	15 (8,5)	0,068
FE, diferença entre linha de base e acompanhamento (%)	0 (11)	-2 (14)	2 (16)	0,052
Gradiente aórtico médio, diferença entre linha de base e acompanhamento (mmHg)	0 (5)	0 (7)	2 (5)	0,212
Gradiente aórtico máximo, diferença entre linha de base e acompanhamento (mmHg)	0 (9)	-2 (9,8)	1 (9)	0,170
Regurgitação aórtica residual moderada/grave	34 (8,0)	2 (5,4)	1 (3,5)	0,540

Resultados descritos por frequência (porcentagem), média ± desvio padrão ou mediana (intervalo interquartil). *ANOVA unilateral, teste não paramétrico de Kruskal-Wallis (variáveis quantitativas) ou teste do qui-quadrado (variáveis categóricas), p < 0,05. AIT: ataque isquêmico transitório, FE: fração de ejeção, NYHA: New York Heart Association, RM: regurgitação mitral, STS: Society of Thoracic Surgeons, TAVI: implante transcateter de válvula aórtica, VE: ventrículo esquerdo.

### Mortalidade

**Figura 3:**
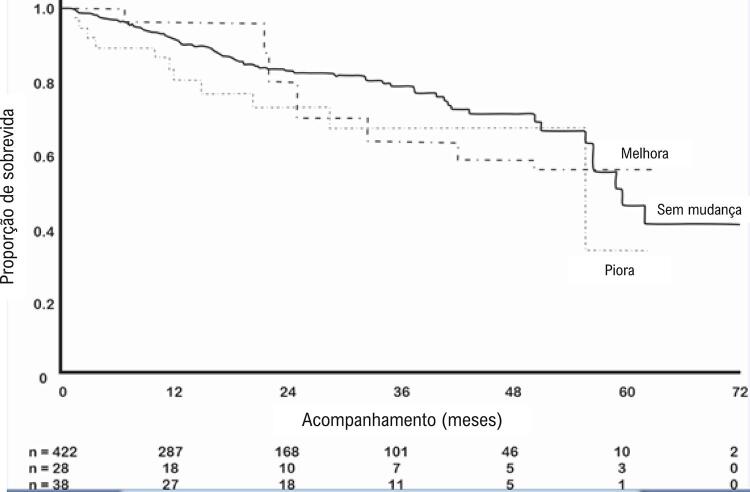
– Curvas de Kaplan-Meier com probabilidades de sobrevida desde a alta até os períodos de acompanhamento para grupos com melhora, sem mudança e piora da regurgitação mitral (RM) após TAVI (n = 488). Modelos de regressão de Cox comparando RM inalterada à piora da RM: p = 0,005; comparando RM inalterada com melhora da RM: p = 0,377.

Mudanças na gravidade da RM na linha de base versus a alta (tanto melhora [HR 1,17; IC 95%, 0,69-1,98; p = 0,56] quanto piora [HR 1,28; IC 95%, 0,70-2,32; p = 0,43]) não foram preditores significativos de mortalidade tardia após TAVI, mesmo quando ajustado para fatores determinantes de sobrevida, como nível basal de hemoglobina (HR 0,89; IC 95%, 0,81-0,98; p = 0,013), insuficiência cardíaca congestiva de classe funcional III/IV da NYHA (HR 1,95; IC 95%, 1,14-3,34; p = 0,015), e valvoplastia aórtica por balão anterior (HR 2,19; IC 95%, 1,29-3,72; p = 0,004). Em uma análise não ajustada, as mudanças tardias na gravidade da RM também não tiveram um impacto nas taxas de mortalidade. No entanto, quando ajustados para fatores que aumentaram a mortalidade neste período, como insuficiência cardíaca congestiva de classe funcional III/IV NYHA (HR 2,6; IC 95%, 1,11-6,05; p = 0,026) e valvoplastia aórtica por balão anterior (HR 2,5; IC 95% , 1,31-4,83; p = 0,005), a piora da RM entre a alta e os períodos de acompanhamento, em comparação com a RM inalterada, foi fortemente associada a um aumento do risco de mortalidade (HR 2,74; IC 95%, 1,36-5,48; p = 0,005) ( Tabela 4 ). As curvas de Kaplan-Meier que demonstram as probabilidades de sobrevida para cada grupo desde a alta até o acompanhamento são mostradas na Figura 3 .

**Tabela 4:** – Impacto de grupos definidos por mudanças na gravidade da RM: linha de base até a alta, alta até acompanhamento, e mortalidade geral

	Tempo médio (meses)	Óbitos (%)	Não ajustado		Ajustado**	

			HR (IC 95%)	valor p*	HR (IC 95%)	valor p*
**RM da linha de base até a alta**						
Sem mudança (referência)	54,6	24,5	1		1	
Piora	44,0	28,6	1,21 (0,68 – 2,14)	0,512	1,28 (0,70–2,32)	0,426
Melhora	35,1	25,0	1,03 (0,61 – 1,73)	0,912	1,17 (0,69–1,98)	0,561
**RM da alta até o acompanhamento**						
Sem mudança (referência)	68,1	16,9	1		1	
Piora	51,3	28,2	1,61 (0,85 – 3,04)	0,141	2,74 (1,36 – 5,48)	0,005
Melhora	50,5	18,8	1,42 (0,62 – 3,29)	0,408	1,48 (0,62 – 3,50)	0,377

*Modelo de regressão de Cox (razão de verossimilhança stepwise e backward) e teste de Wald, p < 0,05. **Disfunção mitral da linha de base até a alta: ajustada para nível basal de hemoglobina, classe funcional da NYHA e valvoplastia aórtica por balão prévia; **Disfunção mitral da alta até o acompanhamento: ajustada para classe funcional da NYHA e valvoplastia aórtica por balão prévia. HR: razão de chances, IC: intervalo de confiança, RM: regurgitação mitral.

## Discussão

No presente estudo, observamos o seguinte: 1) a RMMG na linha de base em pacientes submetidos ao TAVI foi associada à idade, à presença de comorbidades e à gravidade da estenose aórtica; 2) a RMMG na linha de base foi um preditor de mortalidade tardia após TAVI; 3) aproximadamente metade dos pacientes com RMMG na linha de base apresentou melhora da gravidade da RM imediatamente após TAVI e, além disso, 37% dos pacientes com RMMG na alta apresentaram melhora da RM no acompanhamento tardio; 4) a regurgitação aórtica moderada/grave na linha de base foi um preditor de melhora imediata da RMMG após TAVI; 5) os pacientes que apresentaram melhora progressiva da RM no acompanhamento tardio após TAVI foram aqueles que apresentaram menor FEVE na linha de base e melhora da FEVE após a intervenção; e, por fim, 6) a piora progressiva da gravidade da RM no acompanhamento tardio pós-TAVI foi um preditor independente de mortalidade; no entanto, nenhum preditor foi identificado para tal piora.

Em corroboração com outros estudos, 20% dos pacientes do Registro Brasileiro de TAVI apresentavam RMMG na linha de base e esses pacientes tinham comorbidades mais graves do que aqueles com RM menos grave. ^11 , 15 - 19^ No entanto, existe alguma controvérsia na literatura a respeito do valor prognóstico da RMMG basal na mortalidade dos pacientes após TAVI. Alguns estudos não mostraram correlação, ^15 , 18 , 20^ enquanto outras publicações demonstraram a influência significativa da RM na mortalidade precoce e/ou tardia, ^2 , 5 , 16 , 19 - 23^ em particular uma análise do US Transcatheter Valve Therapy Registry que inclui mais de 4.000 pacientes. ^22^ De maneira semelhante a esses estudos posteriores, nossos resultados também demonstraram que a presença da RMMG na linha de base leva ao aumento da taxa de mortalidade tardia após TAVI.

De acordo com a gravidade da RM, havia quatro grupos e eles foram analisados juntos nos grupos de RM ausente/leve e moderada/grave. Isso foi feito devido ao número pequeno de pacientes com RM grave (n = 20 pacientes, 2,4%). Na literatura, todos os estudos relacionados à RM em pacientes com TAVI analisaram RM moderada e grave em apenas um grupo (RM moderada/grave) como nós. ^2 , 3 , 5 , 7 , 15 , 20^

Não foi possível definir a etiologia da RM (orgânica/degenerativa versus funcional) com base em nossos dados de registro. Vollenbroich et al., ^7^ estudou a influência da RM funcional versus degenerativa no desfecho clínico após TAVI, verificando 36% de RM funcional e 64% de RM degenerativa entre os pacientes com RMMG. A RM degenerativa apresentou risco aumentado durante o acompanhamento de longo prazo após TAVI, em relação à RM funcional. Muratori et al., ^3^ também verificou RM orgânica mais prevalente entre pacientes com RMMG submetidos a TAVI, mostrando uma maior redução do grau de RM após TAVI em pacientes com RM funcional e um impacto negativo no acompanhamento de longo prazo para a RM orgânica. Portanto, a etiologia da RM pode influenciar o prognóstico após TAVI, mas não foi possível estudar esse assunto em nossa população de pacientes.

Estão disponíveis poucas informações sobre a frequência e o valor prognóstico das mudanças na gravidade da RM após TAVI. Conforme ilustrado na Figura 2 e, de acordo com os achados de Boerlage-van Dijk et al., ^24^ mais de 80% dos nossos pacientes não apresentaram alteração no seu grau basal de RM durante o acompanhamento tardio após TAVI. No entanto, quase metade dos pacientes com RMMG na linha de base apresentou um grau de RM melhorado imediatamente após TAVI. Entre aqueles sem melhora imediata, quase 40% apresentou melhora no acompanhamento tardio. A literatura recente sugere que a gravidade da RM pré-procedimento melhora após TAVI em 29% a 70% dos pacientes e, na maioria dos casos, é mantida no acompanhamento, tendo um impacto favorável na mortalidade tardia e nas taxas de re-hospitalização após TAVI. ^16 , 19 , 22 - 26^ Como preditores dessa melhora, foram identificados a ausência de calcificação anular mitral, ^17 , 27^ a RM funcional (ao invés da degenerativa), ^6 , 21 , 27^ a ausência de hipertensão pulmonar, ^17 , 21 , 27^ a ausência de fibrilação atrial, ^21 , 24 , 27^ bloqueio de ramo esquerdo persistente, ^27^ gradientes transaórticos iniciais mais altos, ^17^ a ausência de doença arterial coronariana concomitante ^26^ e o implante de uma prótese Edwards-Sapien em vez de CoreValve. ^28^ Identificamos menor FEVE na linha de base e uma melhora na FEVE após a intervenção como preditores de melhora da RM. Estes preditores também foram identificados por outros autores, ^16 , 29 , 30^ podendo ser explicados pelo remodelamento ventricular esquerdo reverso e a consequente redução das forças de alongamento do complexo valvar mitral após TAVI. Esta explicação é apoiada pela demonstração anterior de que os pacientes com melhora da gravidade da RM após TAVI mostram uma redução significativa no volume diastólico final do VE e alterações geométricas anulares mitrais favoráveis após intervenção aórtica. ^31^ A influência da redução do volume diastólico final do VE na melhora da RM também foi demonstrada pela associação de regurgitação aórtica moderada/grave na linha de base com a melhora precoce da gravidade da RM após TAVI, conforme demonstramos.

O Registro Brasileiro de TAVI foi planejado para incluir a maioria dos procedimentos de TAVI realizados no Brasil e, como uma amostra do mundo real, incluímos tanto EAo grave na válvula nativa, que constituía a grande maioria (98%), quanto pacientes com biopróteses aórticas cirúrgicas degeneradas (n = 16, 2% dos pacientes). Isto poderia ser considerado uma falha na nossa seleção de pacientes, mas um estudo recente de Akodad et al. demonstrou que o TAVI válvula-em-válvula é tão seguro e viável quanto o TAVI na EAo nativa, sem influência significativa no acompanhamento destes pacientes. ^32^ Tal achado indica que a inclusão de um pequeno número de biopróteses cirúrgicas degeneradas não deve afetar os nossos resultados e conclusões.

Um dos achados mais importantes do presente estudo foi que a deterioração progressiva da RM tem um impacto negativo na mortalidade tardia em pacientes submetidos a TAVI. Sabe-se que uma porção significativa dos pacientes que apresentam melhora inicial da gravidade da RM, tanto após a troca cirúrgica da válvula aórtica quanto após o TAVI, regride ao estado basal se acompanhados por mais de 1 ano. ^33 , 34^ Porém, foi raramente relatado na literatura o achado de que esta piora da RM é um preditor independente de taxas mais altas de mortalidade tardia. ^25^ Este achado pode desempenhar um papel importante em futuras estratégias terapêuticas durante o acompanhamento para TAVI. A associação entre piora da RM e aumento da mortalidade após TAVI não indica que o tratamento para RM levaria à melhoria da evolução após TAVI, uma vez que pode ser apenas um indicativo da progressão da insuficiência cardíaca. No entanto, o tratamento percutâneo associado para RM já tem sido utilizado para pacientes com TAVI com bons resultados, ^35^ e esta terapia combinada poderá ser uma opção no futuro.

### Limitações

O presente estudo tem algumas limitações. Devido à natureza não randomizada do estudo, não houve grupo controle e, como o desenho do estudo foi observacional, falhas na seleção dos pacientes são possíveis. No entanto, o Registro de TAVI reflete a prática do mundo real no ambiente brasileiro. A análise foi parcialmente baseada em dados retrospectivos e também incluiu coleta prospectiva de dados na maioria dos pacientes. Embora os critérios ecocardiográficos para quantificação da RM tenham sido definidos pelas diretrizes atuais, não existe laboratório central para avaliação ecocardiográfica, podendo, portanto, estar sujeito a variação inter-observador. Os casos de RM foram separados de acordo com a gravidade, mas não foi possível definir a sua etiologia (orgânica versus funcional) com base nos dados do registro. A duração do acompanhamento tardio teve grande variação, uma vez que os pacientes foram incluídos continuamente de 2008 a 2015; desta maneira, alguns pacientes demoraram mais para evidenciar mudanças de remodelação após TAVI. Por fim, uma porção não negligenciável de pacientes foi perdida durante o acompanhamento ecocardiográfico.

## Conclusões

O Registro Brasileiro de TAVI é a maior série de TAVI na América do Sul. Inclui o primeiro procedimento realizado no Brasil e tem o acompanhamento mais longo desses pacientes. O registro reflete a prática do mundo real no ambiente brasileiro. Em nosso estudo, é evidente que a RMMG na linha de base foi um preditor de maior taxa de mortalidade tardia após a intervenção. A maioria dos pacientes com RMMG na linha de base, especialmente aqueles com FEVE mais baixa na linha de base e aqueles que mostraram melhora progressiva na FEVE, mostraram uma melhora no grau de RM durante o acompanhamento. A piora progressiva da gravidade da RM após TAVI resultou em maior taxa de mortalidade tardia e deve ser considerada no atendimento futuro desses pacientes.

## * Material suplementar

Para informação adicional, por favor, clique aqui.
