# Supplementary material for: A Relação entre Regurgitação Mitral e Implante Transcateter de Válvula Aórtica: um Estudo de Acompanhamento Multi-Institucional
Source: Arq Bras Cardiol. 2021 Jun 8;116(6):1059–69. [Article in Portuguese] doi: 10.36660/abc.20190772 (PMC8288547; doi:10.36660/abc.20190772)
Supplement: Supplementary file 1 [file 2019-0772_Appendix.pdf]

Appendix

Participating Centers of the Brazilian TAVI Registry:

1. Hospital Israelita Albert Einstein, São Paulo - SP
2. Hospital de Clínicas da Universidade Federal do Paraná, Curitiba – PR
3. Hospital Pró-Cardíaco, Rio de Janeiro – RJ
4. Instituto de Cardiologia, Porto Alegre - RS
5. Hospital Beneficiência Portuguesa, São Paulo – SP
6. Instituto do Coração, São Paulo – SP
7. Instituto Nacional de Cardiologia, Rio de Janeiro – RJ
8. Pontifícia Universidade Católica, Porto Alegre – RS
9. Hospital S.O.S. Cardio, Florianópolis – SC
10. Hospital Oswaldo Cruz, São Paulo – SP
11. Instituto Dante Pazzanese de Cardiologia, São Paulo – SP
12. Hospital Pilar, Curitiba – PR
13. Hospital Sirio Libanês, São Paulo – SP
14. Hospital Santa Izabel, Salvador – BA
15. Hospital Barra D'Or, Rio de Janeiro – RJ
16. Hospital Federal dos Servidores do Estado, Rio de Janeiro – RJ
17. Hospital Regional do Sul de Minas, Varginha – MG
18. Hospital São Vicente de Paulo, Passo Fundo – RS
19. Santa Casa de Misericórdia, Porto Alegre – RS
20. Clínica São Vicente, Rio de Janeiro – RJ
21. Hospital do Coração, São Paulo – SP
22. Hospital 9 de Julho, São Paulo - SP
