# Supplementary material for: A Relação entre Regurgitação Mitral e Implante Transcateter de Válvula Aórtica: um Estudo de Acompanhamento Multi-Institucional
Source: Arq Bras Cardiol. 2021 Jun 8;116(6):1059–69. [Article in Portuguese] doi: 10.36660/abc.20190772 (PMC8288547; doi:10.36660/abc.20190772)
Supplement: Translation

# Translation: Relationship between Mitral Regurgitation and Transcatheter Aortic Valve Implantation: a Multi-Institutional Follow-up Study

**Authors:** Luciana de Cerjat Bernardes P. da Cunha, Enio Eduardo Guerios, Claudio Leinig Pereira da Cunha, Luiz A. Carvalho, Pedro Lemos, Rogério Sarmento-Leite, Alexandre A. Abizaid, José Antonio Mangione, Adriano Dourado Oliveira, Alexandre Siciliano, Vinicius Esteves, Fábio Sândoli de Brito

**Affiliations:** 1 Brazil Universidade Federal do Paraná - Hospital de Clínicas - UFPR, Curitiba, PR - Brazil; 2 Brazil Hospital Pró-Cardíaco, Rio de Janeiro, RJ - Brazil; 3 Brazil Hospital Israelita Albert Einstein, São Paulo, SP - Brazil; 4 Brazil Instituto de Cardiologia, Porto Alegre, RS - Brazil; 5 Brazil Hospital Beneficência Portuguesa de São Paulo, São Paulo, SP - Brazil; 6 Brazil Hospital Santa Izabel, Salvador, BA - Brazil; 7 Brazil Rede D’Or São Luiz, São Paulo, SP - Brazil; 8 Brazil Universidade de São Paulo Instituto do Coração, São Paulo, SP - Brazil; 9 Brazil Hospital Sírio-Libanês, São Paulo, SP - Brazil

**Keywords:** Aortic Valve Insuffciency, Mitral Valve Insufficiency, Aortic Valve Transcatheter Implantation, Epidemiology, Survival Analysis, Echocardiography/methods

## Abstract

**Background:**

Mitral regurgitation (MR) is prevalent in patients undergoing transcatheter aortic valve implantation (TAVI). There are some controversies about the prognostic impact of MR in survival of TAVI patients.

**Objective:**

To examine the relationship between TAVI and MR in a patient population from the Brazilian TAVI Registry.

**Methods:**

Seven hundred and ninety-five patients from the Brazilian TAVI Registry were divided at baseline, discharge, and follow-up according to their MR grade as follows: absent/mild (AMMR) or moderate/severe (MSMR). They were subsequently regrouped according to their immediate and late changes in MR severity after TAVI as follows: no change, improved, or worsened MR. Predictors and prognostic impact on baseline as well as changes in MR severity were analyzed. Statistical significance was set at p < 0.05.

**Results:**

Baseline MSMR was present in 19.3% of patients and was a predictor of increased late mortality. Immediately after TAVI, 47.4 % of cases improved to AMMR, predicted by a higher Society of Thoracic Surgeons score and a higher grade of baseline aortic regurgitation. Upon follow-up, 9.2% of cases of AMMR worsened to MSMR, whereas 36.8% of cases of MSMR improved to AMMR. Lower baseline left ventricular ejection fraction (LVEF) and improvement in LVEF at follow-up were predictors of MR improvement. Progressive worsening of MR upon follow-up was an independent predictor of higher late mortality after TAVI (p = 0.005).

**Conclusions:**

Baseline MSMR predicts late mortality after TAVI. Lower LVEF and improved LVEF at follow-up predict MR improvement after TAVI. Progressive worsening of MR severity at follow-up is an independent predictor of late mortality, which is a rare finding in the literature.

## Introduction

Approximately two thirds of patients with severe symptomatic aortic stenosis (AS) and indication for surgical valve replacement present with some degree of mitral regurgitation (MR)^1^ and, in some cases, an indication for double valve replacement surgery.^2^ For patients undergoing isolated aortic valve replacement, moderate or severe MR may be associated with higher mortality rates, congestive heart failure, and subsequent mitral valve surgery.^3^

For patients with severe AS and MR for whom surgery is not the ideal therapeutic choice, transcatheter aortic valve implantation (TAVI) may be a suitable option.^1 , 2^ Since, in some patients, a grade reduction may be expected, or subsequent transcatheter mitral valve intervention may be indicated, MR is generally not treated in this scenario.^1 , 4^ However, in the case of isolated aortic surgery, MR severity may decrease, remain unchanged, or even increase after TAVI.^1 , 5^ Although many studies consistently demonstrate that important MR at baseline is associated with poorer outcomes,^4 , 6^ information regarding the prognostic implications of changes in MR severity after TAVI is scarce.^7^

The aim of this study was to examine the relationship between TAVI and MR in a patient population from the Brazilian TAVI Registry.^8^ We hypothesized that moderate/severe MR (MSMR) at baseline and progressive deterioration of MR influences the prognosis of TAVI.

## Methods

### Patients

The multicenter Brazilian TAVI Registry is a voluntary participation registry, conducted since 2008 by the Brazilian Society of Interventional Cardiology, which aggregates the results of TAVI performed in 22 centers across Brazil. Patients have been retrospectively and prospectively included in the registry since the first TAVI was performed in Brazil. The registry was approved by the Ethics Committee of the Albert Einstein Hospital, São Paulo, on November 10, 2010, and inserted in the “ *Plataforma Brasil* ” (a national and unified database of research records involving human beings). All prospectively included patients provided informed written consent.

Indication for TAVI was limited to groups of inoperable or high-surgical-risk patients with severe symptomatic AS or degenerated surgical bioprosthesis. The surgical mortality risk was estimated using the EuroScore^9^ and the Society of Thoracic Surgeons (STS) risk score.^10^ Details, definitions, and partial registry results have been previously published.^8^

This study included patients treated between January 2008 and January 2015. Patients who had previously undergone mitral valve surgery or patients who did not have adequate pre- and post-intervention echocardiographic records were excluded from the analysis. Follow-up was performed at medical visits with echocardiographic studies; the last follow-up echocardiogram was used to compare with baseline and discharge studies.

### TAVI procedure

TAVI was performed using CoreValve prostheses (Medtronic, Minneapolis, MN, USA) by transfemoral and transubclavian access, Sapien XT (Edwards Lifesciences, Irvine, CA, USA) by transfemoral and transapical access, and Inovare (Braile Biomédica, São José do Rio Preto, SP, Brazil) implanted only by the transapical route. The procedure was performed according to standard techniques, previously described in detail.^11 - 13^ The choice of access, type of anesthesia (general or sedation), and the use of intraoperative transesophageal echocardiography was left to the operator’s discretion. After the intervention, aspirin (100 mg once daily) and clopidogrel (300 mg as a loading dose and 75 mg once daily thereafter) were administered to the patients for a minimum of 30 days. A complete transthoracic echocardiogram of the patients was performed in the pre-, peri-, and post-intervention periods (if there were several echocardiograms, the last one was included). MR severity was defined as absent, mild, moderate, or severe according to the recommendations of the American Society of Echocardiography, integrating structural, Doppler, and quantitative parameters.^14^

Patients’ clinical data and echocardiograms were analyzed at baseline, hospital discharge, and late follow-up (mean follow-up time of 16.6 months). In each of these periods, the patients were separated into two groups, according to their MR grade. One group included patients with absent or mild MR (AMMR), and the other included those with MSMR, as described in prior studies.^2 , 15^ Subsequently, patients were regrouped according to the change in MR severity after TAVI when comparing baseline, discharge, and follow-up periods, as follows: patients who showed no change in MR grade, those with worsened MR (from AMMR to MSMR), and those with improved MR severity (from MSMR to AMMR). Clinical and echocardiographic predictors of MR improvement/worsening were identified, and the relationship between changes in MR grade and mortality rates was analyzed.

### Statistical analysis

Statistical analyses were performed with the IBM SPSS Statistics for Windows, Version 20.0 (IBM Corp, Armonk, NY, USA). Continuous variables were expressed as mean and standard deviation or median and range, while categorical variables were expressed as frequencies and percentages. Kolmogorov-Smirnov test was used to verify the normality of the data; normality of data distribution was accepted for most of the variables, without compromising other analyses. Associations in categorical variables between groups were assessed using Pearson’s chi-square test. Continuous variables were analyzed using Student’s t test for independent samples or non-parametric Mann-Whitney test to compare groups defined by baseline MR grade (AMMR or MSMR). One-way analysis of variance (ANOVA) or non-parametric Kruskal-Wallis test was used to compare the groups defined by MR changes (no change, worsened, or improved). Survival probability was estimated by Kaplan-Meier curves. To analyze the effect of MR changes on survival time, non-adjusted and adjusted Cox proportional hazard regression models were adapted by including covariates with p < 0.05 in the non-adjusted models. Final models were assessed by stepwise backward likelihood ratio method considering a p value < 0.05 for inclusion and exclusion criteria. Hazard ratios (HR) and 95% confidence intervals (CI) were presented for the final models. Statistical significance was set at p < 0.05.

## Results

### Baseline characteristics of the patients

Of the 819 patients included in the Brazilian TAVI Registry, 795 patients were included in this analysis. A patient flow diagram is shown in Figure 1 , and Table 1 details patients’ baseline clinical characteristics according to their baseline MR grade. Prior to the procedure, MR was absent/mild in 642 patients (80.7%) and moderate/severe in 153 patients (19.3%). Patients with MSMR were older, and they presented with more comorbidities (renal failure, lower hemoglobin levels, pulmonary hypertension, atrial fibrillation, previous pacemaker implantation, more advanced heart failure grades), higher surgical risk scores, lower ejection fractions, larger LV diastolic diameters, more severe aortic regurgitation, smaller aortic valve areas, and lower aortic gradients.

**Figure 1:** Patient flow diagram. This flow chart specifies the mortality rate in the AMMR and MSMR groups. Note that the mortality rate before discharge includes the peri-procedural mortality. “Incomplete records” are related to the absence of good echocardiograms for analysis. AMMR: absent/mild mitral regurgitation; MSMR: moderate/severe mitral regurgitation; FU: follow-up.

**Table 1:** – Baseline characteristics of patients and comparison of groups defined by baseline MR dysfunction (n = 795)

Characteristic	Whole population (n = 795)	According to baseline MR dysfunction		p value*

		Absent/mild (n = 642)	Moderate/severe (n = 153)	
Age (years)	81.5 ± 7.3	81.2 ± 7.5	83.1 ± 6.6	0.002
Male	389 (48.9)	313 (48.8)	76 (49.7)	0.838
Coronary artery disease	465 (58.4)	375 (58.4)	90 (58.8)	0.926
Previous myocardial infarction	117 (14.7)	99 (15.4)	18 (11.8)	0.251
Peripheral vascular disease	136 (17.1)	118 (18.4)	18 (11.8)	0.051
Stroke/TIA	63 (7.9)	50 (7.8)	13 (8.5)	0.771
Diabetes	253 (31.8)	206 (32.1)	47 (30.7)	0.744
Systemic arterial hypertension	601 (75.5)	484 (75.4)	117 (76.5)	0.780
Renal failure	615 (77,3)	485 (75.5)	130 (85.0)	0.012
Preprocedural pacemaker	81 (10.2)	57 (8.8)	24 (15.6)	0.012
Hemoglobin (mg/dl)	11.8 ± 1.7	11.8 ± 1.8	11.5 ± 1.6	0.045
Pulmonary hypertension	176 (22.1)	133 (20.7)	43 (28.1)	0.048
NYHA functional class III or IV	648 (81.5)	511 (79.6)	137 (89.5)	0.004
Atrial fibrillation	106 (13.3)	78 (12.3)	28 (18.5)	0.044
EuroScore mortality	16 ( 17.6)	15.2 (16.6)	21.1 (17.5)	0.001
STS score mortality	7.2 (10.5)	6.6 (9.9)	10.9 (12)	<0.001
Moderate/severe baseline aortic regurgitation	95 (11.9)	60(10,9)	35(23.0)	<0,001
Previous balloon aortic valvuloplasty	50 (6,2)	36 (5,6)	14 (9,2)	0,105
Baseline EF (%)	58.7 ± 14.9	60.1 ± 14.4	53.2 ± 16.0	<0.001
Baseline LV diastolic diameter (mm)	50.8 ± 9.4	50.2 ± 8.8	53.4 ± 10.3	0.001
Baseline aortic valve area (cm^2^)	0.66 ± 0.19	0.67 ± 0.19	0.63 ± 0.19	0.016
Baseline mean aortic gradient (mmHg)	49.3 ± 16.0	50.1 ± 15.7	46.3 ± 16.5	0.010
Baseline peak aortic gradient (mmHg)	81.0 ± 24.8	82.3 ± 24.6	76.0 ± 25.0	0.005

Results described by frequency (percentage), mean ± standard deviation or median (interquartile range). *Student t test for independent samples, non-parametric Mann-Whitney test (quantitative variables), or chi-square test (categorical variables), p < 0.05. EF: ejection fraction, LV: left ventricle, MR: mitral regurgitation, NYHA: New York Heart Association, STS: Society of Thoracic Surgeons, TIA: transient ischemic attack.

CoreValve prostheses were implanted in 597 patients (73%) by transarterial accesses, Sapien XT in 200 patients (24%) (3 by transapical and 197 by transarterial approaches), and Inovare in 22 patients (3%) by transapical access. In total, there were 770 patients who received the prostheses by transarterial accesses, while 25 were by transapical access. Seven hundred and seventy nine patients (98%) had prostheses for native severe AS, and 16 (2%) had valve-in-valve prosthesis for degenerated surgical bioprostheses.

### Predictors for late mortality

According to the adjusted Cox regression model, peripheral vascular disease (HR 1.6; 95% CI, 1.11-2.32; p = 0.012), previous balloon aortic valvuloplasty (HR 1.97; 95% CI, 1.25-3.11; p = 0.004), and baseline MSMR (HR 1.50; 95% CI, 1.05-2.14; p = 0.027) were independent baseline predictors of late mortality, with mean follow-up time of 16.6 months and median follow-up of 12.4 months (first quartile: 2.6 months and third quartile: 24.7 months) in this population

### Changes in MR severity: pre-intervention versus discharge

After intervention, MR grade was compared between baseline and discharge in a total of 697 patients. TAVI did not change MR grade in comparison with baseline in 83.8% (n = 584) of patients. MR severity worsened after TAVI in 8.7% (n = 49) of patients with baseline AMMR, but it improved in 47.8% (n = 64) of those with baseline MSMR ( Figure 2 ).

**Figure 2:** – Changes in mitral regurgitation (MR) severity: baseline, discharge, and follow-up periods. This includes patients with complete echocardiography data in all three periods. Baseline: n = 795; discharge: n = 697; follow-up: n = 488. **Variation of MR grade when comparing baseline to the last follow-up for the whole population, excluding deaths and incomplete records.

There was a higher prevalence of renal failure in patients whose MR grade worsened after TAVI (p = 0.022). Upon univariate analysis, a higher STS score (p = 0.013) and a more severe baseline aortic regurgitation (p = 0.010) were predictors of an improvement in MR severity. Other baseline echocardiographic data, as well as changes in parameters, such as the left ventricular ejection fraction (LVEF) and aortic gradient between baseline and discharge, were not associated with MR severity improvement or worsening after TAVI ( Table 2 ).

**Table 2:** – Comparison of groups defined by changes in MR severity: baseline versus discharge after TAVI (n = 697)

Characteristic	Changes in MR severity at baseline versus discharge			p value*

	Unchanged n = 584	Worsened n = 49	Improved n = 64	
Age (years)	81.3 ± 7.5	82.4 ± 5.5	81.9 ± 6.7	0.559
Male	294 (50.3)	21 (42.9)	31 (48.4)	0.590
Coronary artery disease	332 (56.8)	35 (71.4)	38 (59.4)	0.136
Previous myocardial infarction	90 (15.4)	7 (14.3)	7 (10.9)	0.629
Peripheral vascular disease	106 (18.2)	10 (20.4)	6 (9.4)	0.184
Stroke/TIA	49 (8.4)	4 (8.2)	5 (7.8)	0.986
Diabetes	187 (32.0)	13 (26.5)	20 (31.2)	0.728
Systemic arterial hypertension	429 (73.5)	40 (81.6)	51 (79.7)	0.279
Renal failure	444 (76.0)	44 (89.8)	55 (85.9)	0.022
Hemoglobin (mg/dl)	11.8 ±1.8	12.0±1.7	11.5±1.5	0.374
Pulmonary hypertension	130 (22.3)	8 (16.3)	17 (26.6)	0.431
NYHA functional class III or IV	471 (80.7)	39 (79.6)	55 (85.9)	0.570
Atrial fibrillation	73 (12.7)	8 (16.3)	10 (15.6)	0.642
EuroScore mortality	15.6 (17)	17.4 (15.7)	21.1 (17.9)	0.124
STS score mortality	6.9 (10.2)	9.5 (14.5)	11.5 (12.1)	0.013
Moderate/severe baseline aortic regurgitation	69 (11.9)	5 (10.9)	16 (25.4)	0.010
Previous balloon aortic valvuloplasty	33 (5,7)	3 (6,1)	7 (10,9)	0,309
Baseline EF (%)	59.2 ± 15.0	55.3 ± 15.5	57.3 ± 14.7	0.160
Baseline LV diastolic diameter (mm)	50.8 ± 9.0	51.2 ± 11.0	52.4 ± 10.0	0.430
Baseline aortic valve area (cm^2^)	0.67 ± 0.19	0.67 ± 0.17	0.63 ± 0.20	0.360
Baseline mean aortic gradient (mmHg)	49.5 ± 16.0	46.3 ± 12.6	49.5 ± 19.2	0.434
Baseline peak aortic gradient (mmHg)	80 (33)	75 (34.5)	78 (37.5)	0.324
Difference baseline-discharge EF (%)	1 (10)	1 (16.3)	3 (10)	0.314
Difference baseline-discharge aortic mean gradient (mmHg)	-39.6 ± 16.1	-39.7 ± 12.9	-37.5 ± 23.0	0.686
Difference baseline-discharge peak aortic gradient (mmHg)	-63.1 ± 24.9	-60.3 ± 22.3	-56.6 ± 34.2	0.174

Results described by frequency (percentage), mean ± standard deviation, or median (interquatile range). *One-way ANOVA, non-parametric Kruskal-Wallis test (quantitative variables), or chi-square test (categorical variables), p < 0.05 EF: ejection fraction, LV: left ventricle, MR: mitral regurgitation, NYHA: New York Heart Association, STS: Society of Thoracic Surgeons, TAVI: transcatheter aortic valve implantation, TIA: transient ischemic attack .

### Changes in MR severity: discharge versus follow-up

After discharge, clinical and echocardiographic follow-up was performed in 488 patients, with a mean follow-up time of 16.6 ± 14.1 months (median follow-up: 12.4 months, first quartile: 2.6 months and third quartile: 24.7 months). Compared with discharge, there were no changes in MR severity in 86.4% (n = 422) of patients. Only 9.2% (n = 38) of patients with AMMR at discharge presented with worse MR severity grades, whereas 36.8% (n = 28) of patients with MSMR at discharge presented with an improvement to AMMR at follow-up ( Figure 2 ).

Lower baseline LVEF (p = 0.015) was a predictor of late improvement of MR severity in the univariate analysis. In addition, a strong trend towards late improvement of MR severity was observed in patients with LVEF improvement upon follow-up (p = 0.052, Table 3 ). No predictive factors of late worsening of MR severity were identified.

**Table 3:** – Comparison of groups defined by changes in MR severity: discharge after TAVI versus follow-up periods (n = 488)

Characteristic	Changes in MR severity at discharge versus follow-up (mean = 16.6 months)			p value*

	Unchanged n = 422	Worsened n = 38	Improved n = 28	
Age (years)	81.1 ± 7.3	81.7 ± 6.4	83.9 ± 6.6	0.119
Male	216 (51.2)	15 (39.5)	13 (46.4)	0.356
Coronary artery disease	238 (56.4)	25 (65.8)	19 (67.9)	0.287
Previous myocardial infarction	61 (14.5)	7 (18.4)	6 (21.4)	0.538
Peripheral vascular disease	73 (17.3)	6 (15.8)	6 (21.4)	0.830
Stroke/TIA	27 (6.4)	4 (10.5)	2 (7.1)	0.659
Diabetes	128 (30.3)	13 (34.2)	10 (35.7)	0.755
Systemic arterial hypertension	306 (72.5)	27 (71.1)	21 (75.0)	0.938
Renal failure	323 (76.5)	30 (78.9)	26 (92.9)	0.131
Hemoglobin (mg/dl)	11.8 ± 1.7	11.8 ± 1.7	11.8 ± 2.0	0.968
Pulmonary hypertension	85 (20.1)	9 (23.7)	11 (39.3)	0.055
NYHA functional class III or IV	347 (82.2)	28 (73.7)	24 (85.7)	0.365
Atrial fibrillation	50 (12.0)	7 (18.4)	5 (17.9)	0.407
EuroScore mortality	15.2 (15.8)	19.8 (20)	18.4 (21.2)	0.077
STS score mortality	7 (10.7)	10.9 (13.2)	10.6 (8.2)	0.254
Moderate/severe baseline aortic regurgitation	54 (13.1)	6 (16.2)	3 (11.1)	0.825
Previous balloon aortic valvuloplasty	28 (6,6)	3 (7,9)	1 (3,6)	0,744
Baseline EF (%)	58.6 ± 15.3	59.0 ± 14.5	49.8 ± 16.5	0.015
Baseline LV diastolic diameter (mm)	50,6 ± 8,0	51.4 ± 9.0	51.8 ± 8.0	0.569
Baseline aortic valve area (cm^2^)	0.66 ± 0.19	0.70 ± 0.14	0.62 ± 0.23	0.317
Baseline mean aortic gradient (mmHg)	50.5 ± 16.3	46.0 ± 14.4	45.7 ± 14.4	0.104
Discharge EF (%)	60.4 ± 13.4	61.4 ± 12.7	55.3 ± 15.3	0.117
Discharge LV diastolic diameter (mm)	50.4 ± 9.0	51.8 ± 9.0	51.6 ± 8.0	0.642
Discharge mean aortic gradient (mmHg)	10.2 ± 6.1	9.2 ± 7.9	7.6 ± 3.7	0.131
Discharge peak aortic gradient (mmHg)	18 (11)	15.5 (12.5)	15 (8.5)	0.068
Difference baseline-follow-up EF (%)	0 (11)	-2 (14)	2 (16)	0.052
Difference baseline-follow-up mean aortic gradient (mmHg)	0 (5)	0 (7)	2 (5)	0.212
Difference baseline-follow-up peak aortic gradient (mmHg)	0 (9)	-2 (9.8)	1 (9)	0.170
Moderate/severe residual aortic regurgitation	34 (8.0)	2 (5.4)	1 (3.5)	0.540

Results described by frequency (percentage), mean ± standard deviation, or median (interquatile range). *One-way ANOVA, non-parametric Kruskal-Wallis test (quantitative variables), or chi-square test (categorical variables), p < 0.05 EF: ejection fraction, LV: left ventricle, MR: mitral regurgitation, NYHA: New York Heart Association, STS: Society of Thoracic Surgeons, TAVI: transcatheter aortic valve implantation, TIA: transient ischemic attack.

### Mortality

Changes in MR severity at baseline versus discharge (both improvement [HR 1.17; 95% CI, 0.69–1.98; p = 0.56] or worsening [HR 1.28; 95% CI, 0.70–2.32; p = 0.43]) were not significant predictors of late mortality after TAVI, even when adjusted for survival determining factors such as baseline hemoglobin level (HR 0.89; 95% CI, 0.81-0.98; p = 0.013), NYHA functional class III/IV congestive heart failure (HR 1.95; 95% CI, 1.14-3.34; p = 0.015), and previous balloon aortic valvuloplasty (HR 2.19; 95% CI, 1.29-3.72; p = 0.004). In a non-adjusted analysis, late changes in MR severity also did not impact mortality rates. However, when adjusted for factors that increased mortality in this period, such as NYHA functional class III/IV congestive heart failure (HR 2.6; 95% CI, 1.11-6.05; p = 0.026) and previous balloon aortic valvuloplasty (HR 2.5; 95% CI, 1.31-4.83; p = 0.005), the worsening of MR between discharge and follow-up periods, compared to unchanged MR, was strongly associated with an increased mortality risk (HR 2.74; 95% CI, 1.36-5.48; p = 0.005) ( Table 4 ). Kaplan-Meier curves demonstrating survival probabilities for each group from discharge to follow-up are shown in Figure 3 .

**Table 4:** – Impact of groups defined by changes in MR severity: baseline to discharge, discharge to follow-up, and general mortality

	Mean time (months	Deaths (%)	Non-adjusted		Adjusted**	

			HR (95% CI)	p value*	HR (95% CI)	p value*
**MR from baseline to discharge**						
Unchanged (reference)	54.6	24,5	1		1	
Worsening	44.0	28.6	1.21 (0.68 – 2.14)	0.512	1.28 (0.70–2.32)	0.426
Improvement	35.1	25.0	1.03 (0.61 – 1.73)	0.912	1.17 (0.69–1.98)	0.561
**MR from discharge to follow-up**						
Unchanged (reference)	68.1	16.9	1		1	
Worsening	51.3	28.2	1.61 (0.85 – 3.04)	0.141	2.74 (1.36 – 5.48)	0.005
Improvement	50.5	18.8	1.42 (0.62 – 3.29)	0.408	1.48 (0.62 – 3.50)	0.377

*Cox regression model (stepwise backward likelihood ratio ) and Wald test, p < 0.05. **Baseline-to-discharge mitral dysfunction: adjusted for baseline hemoglobin level, NYHA functional class and previous balloon aortic valvuloplasty; **Discharge-to-follow-up mitral dysfunction: adjusted for NYHA functional class and previous balloon aortic valvuloplasty. CI: confidence interval, HR: hazard ratio, MR: mitral regurgitation.

**Figure 3:** – Kaplan-Meier curves with survival probabilities from discharge to follow-up periods for groups with improvement, no change and worsening of mitral regurgitation (MR) after TAVI (n = 488). Cox Regression Models comparing Unchanged MR to worsening of MR: p = 0.005; comparing unchanged MR to improvement of MR: p = 0.377.

## Discussion

In the present study, we observed the following: 1) baseline MSMR in patients undergoing TAVI was associated with age, the presence of comorbidities, and the severity of aortic stenosis; 2) baseline MSMR was a predictor of late mortality after TAVI; 3) approximately half of the patients with baseline MSMR presented with improved MR severity immediately after TAVI, and, in addition, 37% of patients with MSMR upon discharge presented with improved MR at the late follow-up; 4) baseline moderate/severe aortic regurgitation was a predictor of immediate improvement of MSMR after TAVI; 5) patients who showed a progressive improvement in MR at the late follow-up after TAVI were those who presented with a lower baseline LVEF and improved LVEF after intervention; and, finally, 6) progressive worsening of MR severity at the late follow-up post-TAVI was an independent predictor of mortality; however, no predictor of this worsening was identified.

In corroboration with other studies, 20% of patients in the Brazilian TAVI Registry presented with baseline MSMR, and these patients had more serious comorbidities than those with less severe MR.^11 , 15 - 19^ However, there is some controversy in the literature concerning the prognostic value of baseline MSMR on patient mortality after TAVI. Some studies showed no correlation,^15 , 18 , 20^ whereas other publications demonstrated the influence of significant MR on early and/or late mortality,^2 , 5 , 16 , 19 - 23^ in particular an analysis of the US Transcatheter Valve Therapy Registry comprising more than 4,000 patients.^22^ Similar to these later studies, our results also demonstrated that the presence of MSMR at baseline leads to increased late mortality rate after TAVI.

According to the severity of MR, there were four groups and they were analyzed together in absent/mild MR and moderate/severe MR groups. This was done due to the small number of patients with severe MR (n = 20 patients, 2.4%). In the literature, all the studies related to MR in TAVI patients have analyzed moderate and severe MR in only one group (moderate/severe MR) as we did.^2 , 3 , 5 , 7 , 15 , 20^

The etiology of MR (organic/degenerative versus functional) could not be defined based on our registry data. Vollenbroich et al.^7^ studied the influence of functional versus degenerative MR on clinical outcome after TAVI. They found 36% functional and 64% degenerative MR among the patients with MSMR. Degenerative MR presented increased risk during long-term follow-up after TAVI, in relation to functional MR. Muratori et al.^3^ also found organic MR more prevalent among patients with MSMR who underwent TAVI. They showed a greater reduction of MR degree after TAVI in functional MR and a negative impact on long-term follow-up for organic MR. Thus, the etiology of MR may influence prognosis after TAVI but we could not study this topic in our population of patients.

Little information is available regarding the frequency and prognostic value of changes in MR severity after TAVI. As depicted in Figure 2 , and, in agreement with the findings of Boerlage-van Dijk et al.,^24^ more than 80% of our patients presented with no change in their baseline MR grade at the late follow-up after TAVI. However, almost half of the patients with baseline MSMR presented with an improved MR grade immediately after TAVI. Among those without immediate improvement, almost 40% improved at the late follow-up. Recent literature has suggested that pre-procedure MR severity improves after TAVI in 29% to 70% of patients, and, in most cases, it is sustained at follow-up, having a favorable impact on late mortality and re-hospitalization rates after TAVI.^16 , 19 , 22 - 26^ The absence of mitral annular calcification,^17 , 27^ functional (rather than degenerative) MR,^6 , 21 , 27^ absence of pulmonary hypertension,^17 , 21 , 27^ absence of atrial fibrillation,^21 , 24 , 27^ persistent left bundle branch block,^27^ higher initial transaortic gradients,^17^ absence of concomitant coronary artery disease,^26^ and the implantation of an Edwards-Sapien rather than CoreValve prosthesis^28^ were identified as predictors of this improvement. We identified lower LVEF at baseline and an improvement in LVEF after the intervention as predictors of MR improvement. These predictors have also been identified by other authors,^16 , 29 , 30^ and they can be explained by reverse left ventricular remodeling and the consequent reduction in the mitral valve complex stretching forces after TAVI. This explanation is supported by the previous demonstration that patients with improved MR severity after TAVI show a significant reduction in LV end-diastolic volume and favorable mitral annular geometric changes after aortic intervention.^31^ The influence of reduced LV end-diastolic volume on the improvement of MR was also demonstrated by the association of moderate/severe baseline aortic regurgitation with early improvement of MR severity after TAVI that we demonstrated.

The Brazilian TAVI Registry was planned to include most of the TAVI procedures performed in Brazil, and, as a real-world sample, we included both severe AS in native valve, which constituted the vast majority (98%) and patients with degenerated surgical aortic bioprostheses (n = 16, 2% of patients). This could be considered a flaw in our patient selection, but a recent study by Akodad et al. has shown that valve-in-valve TAVI is as safe and feasible as TAVI in native AS, with no significant influence in the follow-up of such patients.^32^ This finding indicates that the inclusion of a small number of degenerated surgical bioprostheses should not affect our results and conclusions.

One of the most important findings in the present study was that progressive deterioration of MR has a negative impact on late mortality in patients undergoing TAVI. It is known that a significant portion of the patients who show an initial improvement in MR severity, both after surgical aortic valve replacement and after TAVI, regress to baseline status if followed for more than 1 year.^33 , 34^ However, the finding that this MR worsening is an independent predictor of higher late mortality rates has seldom been reported in the literature.^25^ This finding could play an important role in future therapeutic strategies during TAVI follow-up. The association between MR worsening and increased mortality after TAVI does not indicate that MR treatment would lead to improved evolution after TAVI, since it can only be an indication of heart failure progression. However, associated percutaneous MR treatment has already been used for TAVI patients with good results,^35^ and this combined therapy could be an option in the future.

### Limitations

The present study has some limitations. Due to the non-randomized nature of the study, there was no control group, and, as the study design was observational, flaws in patient selection are possible. However, the TAVI Registry reflects the real-world practice in the Brazilian environment. The analysis was partially based on retrospective data and also included prospective data collection in most patients. Although echocardiographic criteria for MR quantification were defined by current guidelines, there is no core lab for echocardiographic evaluation, and it may, therefore, be subject to inter-observer variation. The MR cases were separated according to severity, but their etiology (organic versus functional) could not be defined based on the registry data. The duration of late follow-up had a large variation, since patients were continuously included from 2008 to 2015; thus, some patients took longer to experience remodeling changes after TAVI. Finally, a non-negligible portion of patients was lost during echocardiographic follow-up.

## Conclusions

The Brazilian TAVI Registry is the greatest series of TAVI in South America. It includes the first procedure carried out in Brazil, and it has the longest follow-up of such patients. The TAVI Registry reflects the real-world practice in the Brazilian environment. From our study, it is evident that baseline MSMR was a predictor of a higher late mortality rate after intervention. Most of the patients with baseline MSMR, especially those with a lower baseline LVEF and those who showed progressive improvement in LVEF, showed an improved MR grade at the follow-up. Progressive worsening of MR severity after TAVI resulted in a higher late mortality rate, and it should be considered in the future care of these patients.

## * Supplemental Materials

For additional information, please click here.
